# Effects of Psychotropic Medications Taken by Pregnant Women on Newborn Medical Condition and Lactation Method

**DOI:** 10.1002/npr2.70005

**Published:** 2025-01-30

**Authors:** Yuta Yoshino, Toru Yagi, Koichi Tsubouchi, Yusuke Takaishi, Yuki Ozaki, Jun‐ichi Iga, Keiichi Matsubara, Yuko Matsubara, Yuka Uchikura, Takashi Sugiyama, Shu‐ichi Ueno

**Affiliations:** ^1^ Department of Neuropsychiatry, Molecules and Function Ehime University Graduate School of Medicine Toon Ehime Japan; ^2^ Department of Obstetrics & Gynecology Ehime University School of Medicine Toon Ehime Japan

## Abstract

**Background:**

Maternal psychiatric condition during the perinatal period is relevant to children's cognitive development and mental health. Psychotropic medications are necessary to maintain the mental health of pregnant women with psychiatric disorders, but they are often avoided due to concerns about adverse effects, such as congenital malformations and abnormal neurodevelopment. A retrospective study of pregnant women with psychiatric disorders using psychotropic medications was performed to clarify maternal and child demographic data and to investigate whether psychotropic medications affected the Apgar score and the decision to breastfeed.

**Methods:**

Data of pregnant women with psychiatric disorders who were referred from the Department of Obstetrics and Gynecology to the Department of Neuropsychiatry at Ehime University Hospital from January 2014 to December 2022 were collected retrospectively. Pearson's chi‐squared test and multiple regression analysis were used for statistical analyses.

**Results:**

A total of 226 women were included; 194 gave birth at our hospital, of whom 79 (40.7%) were taking psychotropic drugs at the time of delivery. None of the children had malformations. There was no relationship between the use of psychotropic medications and the choice to breastfeed. Multiple regression analysis showed that only the gestational weeks at birth were significantly associated with birth weight (*p* < 0.001) and Apgar score (1 min: *p* = 0.030; 5 min: *p* = 0.044).

**Conclusions:**

The use of psychotropic medications during the perinatal period appears safe and beneficial for both pregnant women with psychiatric disorders and their children, and breastfeeding should be considered even if the mother continues to take the medication. To clarify these points, prospective studies using large samples from several countries are needed.

## Abstract 2

Psychotropic meds during pregnancy are safe for mothers with psychiatric disorders & their babies. No link to malformations or breastfeeding choice. Birth weight & Apgar scores were tied to gestational weeks, not meds. Larger studies needed.

## Graphical abstract

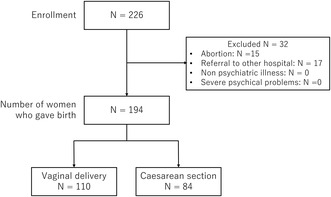

## 1 Introduction

Women of reproductive age, including those in the postpartum period, are prone to psychiatric disorders, and psychotropic medications are necessary to manage their psychiatric condition [1, 2, 3]. Pregnant women with psychiatric disorders tend to have perinatal complications such as stillbirth, premature birth, and low birth weight [4, 5, 6, 7]. Furthermore, psychiatric symptoms during the prenatal period have been reported to affect children's cognitive development and mental health [8, 9]. Traditionally, malformations have been a general concern in women who take psychotropic medications that cross the placenta [10]. Though antiepileptic drugs such as carbamazepine and valproate increase the possibility of congenital malformations [11, 12, 13], most psychotropic medications including antipsychotics, benzodiazepines, and antidepressants have been reported not to increase the risk of congenital malformations [14, 15, 16]. Unless adequate treatment for psychiatric disorders is provided, adverse perinatal outcomes including fetal distress, preterm delivery, and disordered attachment may occur [17, 18]. In terms of complication pregnancy such as hypertensive disorders of pregnancy (HDP) and gestational diabetes mellitus (GDM), maternal psychiatric symptoms are relevant to HDP [19], and olanzapine and clozapine possibly increased risk of GDM [20].

In terms of lactation, evidence shows that breastfeeding is the recommended method of nutrition for children during the first 6 months, because it is associated with a lower risk of infections, obesity, type II diabetes mellitus, and atopic diseases, and with better health in children [21, 22, 23]. In addition, breastfeeding is beneficial for mothers in terms of faster postpartum weight loss, fewer postpartum hemorrhagic complications, and decreased risk of ovarian cancer and metabolic syndrome [21, 24]. Growing evidence has shown that most psychotropic medications are well tolerated by children [25]. However, the decision to continue psychotropic medications during pregnancy and lactation is the mother's decision, and this decision may be based more on the mother's feelings about the medications than on the evidence.

A retrospective study of pregnant women with psychiatric disorders was conducted to clarify the data of mothers and children with maternal use of psychotropic medications and to investigate whether psychotropic medications affected the Apgar score and the decision to breastfeed in our hospital.

## 2 Methods

### 2.1 Study Design

The data of pregnant women referred from the Department of Obstetrics and Gynecology to the Department of Neuropsychiatry at Ehime University Hospital from January 2014 to December 2022 were collected retrospectively. The inclusion criteria for statistical analysis were all pregnant women who (1) were diagnosed as psychiatric illness by two expert psychiatrists according to the 10th revision of the International Classification of Diseases (ICD‐10) and (2) gave birth at our hospital. The exclusion criteria were those who (1) did not gave birth at our hospital, (2) did not diagnosed as psychiatric illness, and (3) had severe psychical problems during pregnant period. This retrospective study was conducted according to the ethics committee of Ehime University Graduate School of Medicine (No. 2411002).

### 2.2 Statistical Analysis

Statistical analysis was conducted using SPSS 22.0 (IBM Japan, Tokyo, Japan). The association between medications and the decision to breastfeed was analyzed using Pearson's chi‐squared test. Multiple regression analyses for birth weight and Apgar score (1/5 min) were conducted with independent factors (maternal age at birth, gestational weeks at birth, alcohol consumption, smoking status, and medication at birth). Simple linear regression analysis was also conducted with those independent factors as sensitivity analysis. Significance was set at the 95% level (*p* = 0.05).

## 3 Results

### 3.1 Demographic Data of Pregnant Women

**FIGURE 1:**
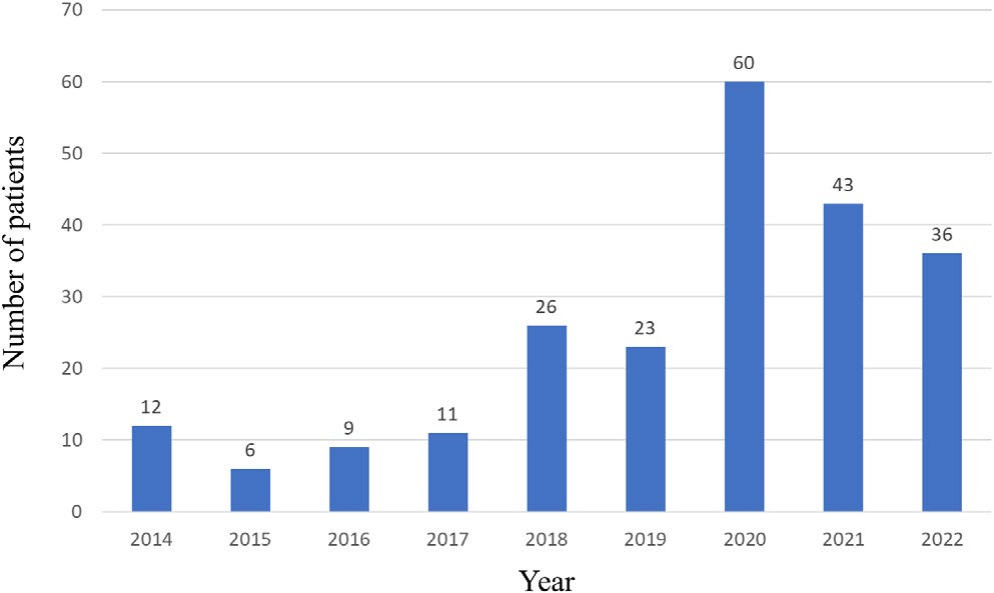
Annual number of pregnant women referred from the Department of Obstetrics and Gynecology to the Department of Neuropsychiatry.

**FIGURE 2:**
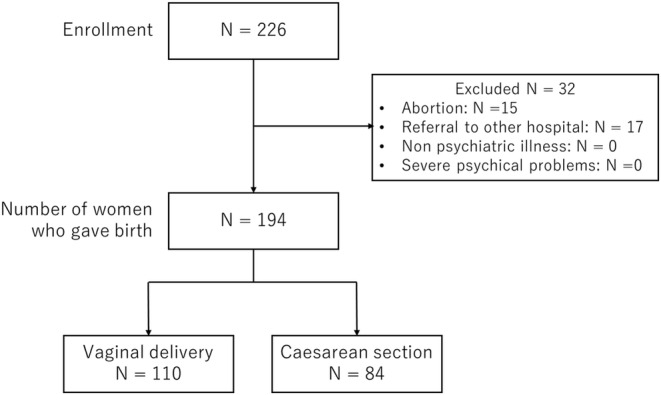
Flow diagram of recruitment process.

The annual number of referred women is shown in Figure 1. Of those, the analysis was conducted on 194 patients who gave birth at our hospital (Figure 2). The demographic data of the 194 pregnant women referred to the Department of Neuropsychiatry are shown in Table 1. Using the ICD‐10 diagnostic criteria, F2, F3, and F4 accounted for 77.9%. HDP and GDM were confirmed in both 7 subjects (6.7%). A total of 194 women gave birth (110 by natural delivery and 84 by cesarean section), and primary cesarean section was 57 (29.4%). Seventy of them (40.7%) were taking psychotropic drugs at the time of delivery. Only six women were admitted to the psychiatric ward at the time of delivery due to a deterioration of their psychiatric symptoms; their details are shown in Table 2.

**TABLE 1:** Demographic data of pregnant women.

Number of subjects	194	
Diagnosis (ICD‐10)	F0: 1	F5: 8
	F1: 0	F6: 6
	F2: 30	F7: 7
	F3: 57	F8: 7
	F4: 66	F9: 2
	Other: 10	
Age when consulting to neuropsychiatry (y.o.)	31.4 ± 5.8 (19–43)	
Alcohol consumption		
1: None	1: 157	
2: Current	2: 2	
3: Past	3: 25	
4: Unknown	4: 10	
Smoking status		
1: None	1: 125	
2: Current	2: 22	
3: Past	3: 38	
4: Unknown	4: 9	
Weeks of pregnancy when consulting to neuropsychiatry (week)	20.9 ± 8.6 (0–39)	
Complication during pregnancy		
1: Hypertensive disorders of pregnancy	1: 13/194 (6.7%)	
2: Gestational diabetes mellitus	2: 13/194 (6.7%)	
Mode of delivery		
1: Vaginal delivery	1: 110	
2: Cesarean section	2: 84	
Reasons for cesarean section	*N* = 84 (43.3%)	
1: Patient's request	1: 10	
2: Due to worse psychiatric symptoms	2: 8	
3: Due to obstetrical indication	3: 39	
4: Previous cesarean pregnancy	4: 27	
Number of medicated cases by psychotropic drugs when birth	70/194 (40.7%)	
Type of medications	Antidepressants, antipsychotics, benzodiazepines, antiepileptic drugs, Chinese medicines	
Hospitalization ward when birth		
1: Obstetrics and gynecology ward	1: 188	
2: Psychiatric ward	2: 6	

Abbreviation: ICD, International Classification of Diseases.

**TABLE 2:** Detailed information of 6 pregnant women who were admitted to the psychiatric ward at delivery.

#	Age (years)	Diagnosis	Symptoms	Birth week	Method of birth	Birth weight (g)
1	19	Organic mental disorder	Irritability, agitation	37	Cesarean section	2360
2	38	Intellectual disability	Possibility of self‐harm	37	Cesarean section	2414
3	31	Bipolar disorder	Agitation, self‐harm	38	Cesarean section	2346
						2218
4	28	Generalized anxiety disorder	Fear of cesarean section, suicidal ideation	37	Natural birth	2590
5	32	Anorexia nervosa	Low body weight, distorted body image, fear of weight gain, self‐induced vomiting	40	Natural birth	2615
6	43	Major depressive disorder	Depressive mood, anxiety, excessive guilt, suicidal ideation	40	Natural birth	3214

### 3.2 Demographic Data of Infant

The demographic data of the children are shown in Table 3. Three sets of twins were identified. At our hospital, all newborns born to mothers taking psychotropic medications at the time of delivery were admitted to the Neonatal Intensive Care Unit (NICU). In fact, 152 of 197 children (77.2%) were admitted to the NICU. The mean Apgar scores at 1 and 5 min were 7.6 ± 1.1 and 8.8 ± 0.5, respectively. There were 11 cases and 0 cases of children with Apgar scores of < 5 at 1 and 5 min, respectively. None of the children had malformations.

**TABLE 3:** Demographic data of infants.

Number of infants	197		
Number of twin births	3/194		
Gestational weeks at birth	38.1 ± 2.0 (27–41)		
Sex of children (male: female)	88: 109		
Birth weight (g)	2865.8 ± 518.7 (762.0–4115.0)		
Apgar score at 1 min/5 min	Score	1 min	5 min
	0–5	11	0
	5–10	186	197
Number of admissions to NICU	152/197 (77.2%)		
Feeding method			
1: Breastfeeding	1: 146		
2: Formula feeding	2: 51		

Abbreviation: NICU, Neonatal Intensive Care Unit.

### 3.3 Differences of Demographic Data of Pregnant Women and Infant With or Without Psychotropic Medications

The differences are shown in Table 4. There seem not to be big differences in each item with or without psychotropic medications.

**TABLE 4:** Demographic data of pregnant women and children based on with or without psychotropic medications.

Pregnant women				
	Non‐medication		Medication	
Number of subjects	124		70	
Diagnosis (ICD‐10)	F0: 1		F0: 0	
	F1: 0		F1: 0	
	F2: 20		F2: 10	
	F3: 29		F3: 28	
	F4: 44		F4: 22	
	F5: 7		F5: 1	
	F6: 4		F6: 2	
	F7: 5		F7: 2	
	F8: 5		F8: 2	
	F9: 2		F9: 0	
	Other: 7		Other: 3	
Complication during pregnancy				
1: Hypertensive disorders of pregnancy	1: 10		1: 3	
2: Gestational diabetes mellitus	2: 9		2: 4	
Mode of delivery				
1: Vaginal delivery	1: 69		1: 41	
2: Cesarean section	2: 55		2: 29	
Children				
Number of children	127		70	
Number of twins	3/124		0/70	
Weeks of birth (week)	37.9 ± 2.2 (27–41)		38.6 ± 1.5 (32–41)	
Birth weight	2788.4 ± 549.6 (762.0–4115.0)		3006.1 ± 426.2 (1310.0–4088.0)	
Apgar score at 1 min/5 min	1 min	5 min	1 min	5 min
0–5	7	0	4	0
5–10	120	127	66	70
Number of admissions to NICU	93/127 (73.2%)		52/70 (74.3%)	
Nutrition method				
1: Breast feeding	1: 94		1: 52	
2: Formula feeding	2: 32		2: 18	

Abbreviations: ICD, International Classification of Diseases; NICU, Neonatal Intensive Care Unit.

### 3.4 Apgar Scores and Decision to Breastfeed With or Without Psychotropic Medications

There was no relationship between the use of psychotropic medications and the decision to breastfeed (*p* = 0.961, Table 5). Multiple regression analysis indicated that, as shown in Table 6, only the birth week was significantly associated with birth weight (*p* < 0.001) and Apgar score (1 min: *p* = 0.030; 5 min: *p* = 0.044). Furthermore, medication was not significantly associated with birth weight (*p* = 0.122) and Apgar score (1 min: *p* = 0.932; 5 min: *p* = 0.668). As a result of simple linear regression analysis, birth week was significantly associated with birth weight (*p* < 0.001) and Apgar score (1 min: *p* = 0.009; 5 min: *p* < 0.001). Other parameters (age at birth, alcohol consumption, smoking status, medication) were not associated with birth weight, Apgar score 1 min and 5 min.

**TABLE 5:** The association between medication and decision on breast feeding.

		Breast feeding	Formula feeding	*p*
Medication	+	52	18	0.961
	−	94	32	

**TABLE 6:** Multiple regression analysis for birth weight and Apgar score (1/5 min).

	Birth weight		Apgar 1 min score		Apgar 5 min score	
	*β*	*p*	*β*	*p*	*β*	*p*
Maternal age at birth	−0.015	0.791	−0.062	0.396	−0.085	0.243
Gestational weeks at birth	0.643	< 0.001	0.238	0.001	0.226	0.002
Alcohol consumption	0.085	0.163	−0.101	0.202	−0.022	0.781
Smoking status	−0.007	0.907	0.070	0.368	−0.033	0.676
Medication	0.088	0.122	−0.006	0.932	0.032	0.668

## 4 Discussion

This retrospective study collected data of 226 pregnant women with psychiatric disorders, examined the demographic data of the mothers and their newborn infant, and investigated whether psychotropic medications affected the Apgar score and the decision to breastfeed. To our knowledge, decision‐making for the method of breastfeeding based on psychotropic drugs from the real word is a new insight. The overall results showed that psychotropic medications were not related to the Apgar score or the decision to breastfeed, and none of the children had malformations.

Overall, the number of referred pregnant women who have psychiatric illnesses increased annually. We think that the increased total number of psychiatric patients affects this result [26]. Furthermore, the environment of social participants for psychiatric patients is gradually improving, it may also affect the increase of the number of patients [27]. The higher rate of primary cesarean section 57/194 (29.4%) is notable when considering the 18.6% of cesarean delivery rates in general Japanese population [28]. Psychiatric and physical reasons possibly elevated cesarean section rate. In terms of major complications during pregnancy, the incidence of HDP and GDM (13/194 [6.7%] and 13/194 [6.7%], respectively) is similar to that in the general Japanese population (8.4% and 6.6%, respectively) [29]. According to the clinical practice guideline [30], the relative risk of cerebral palsy is clearly increased when the Apgar score after 5 min is < 5, and the degree of abnormality correlates with the risk of cerebral palsy. The Apgar score after 1 min was < 5 in 11 children, but none of the children had a score of < 5 after 5 min. Several researchers have reported that low Apgar scores are associated with administration of benzodiazepines [31] and antidepressants [32]. In contrast, Hizkiyahu et al. (2010) conducted a multivariable analysis of both schizophrenia and schizoaffective disorders and concluded that pharmacological treatment was not a risk factor for low Apgar scores or birth weight [33]. These inconsistent results may be due to multiple factors, including not only diagnosis and psychotropic drugs, but also the level of medical care and income disparities in each country. At least so far, in our hospital, low Apgar scores and birth weight were associated with premature birth, but not with psychotropic medications. Furthermore, the average birth weight (2865 g) is similar to the general population's birth weight (male: 3050 g, female: 2960 g) [26].

Recent evidence generally shows that pregnant women can safely continue taking psychotropic medications in most situations [34, 35]. In addition, breastfeeding is beneficial for both the mother [21, 24] and the child [21, 22, 23]. However, in actual clinical situations, it is understandable that mothers are afraid of continuing to take psychotropic medications despite the evidence. Surprisingly, psychotropic medications were not associated with the decision to breastfeed. In our hospital, psychiatrists and obstetricians actively promote breastfeeding based on the accumulation of positive evidence for the child, even though the mother continues to use psychotropic medications [36, 37]. Usually, we answer these points when patients or family ask us. Considering that none of the children had an Apgar score of < 5 at 5 min, breastfeeding with the maternal use of psychotropic medications is relatively safe and beneficial to build a good mother–child relationship.

Six women gave birth after being admitted to a psychiatric ward due to worsening psychiatric symptoms. Worsening psychiatric symptoms and the onset of psychiatric disorders in mothers during the postpartum period are well known [38], but pregnant women before childbirth are also at risk of worsening psychiatric symptoms [39]. Maintaining a good psychiatric condition in the prenatal period can sometimes be difficult. However, since several psychiatric symptoms based on each disease are related to preterm birth and low birth weight, psychiatrists should make efforts to maintain these pregnant women in good psychiatric condition [40]. Those interactions were explained by not only physical/psychiatric conditions, but also genetic risk factors [41]. Further, recent research has shown that early life stress, including prenatal stress, is associated with autism spectrum disorder (ASD) and attention deficit hyperactivity disorder (ADHD) in offspring [42]. In fact, two mothers had preterm births (< 37 weeks), and four children had low birth weights (< 2500 g). Although many factors affect neurodevelopmental disorders, such children should be carefully monitored for developmental abnormalities.

There are several limitations in this retrospective study. First, the relatively small sample size makes it difficult to draw conclusions about the effects of psychotropic medications on the decision to breastfeed, as well as on birth weight and the Apgar score. Furthermore, we are unable to conduct statistical analysis about drug dosages and duration of use. Second, the mechanisms that may affect the course of neurodevelopment are unclear. Lastly, this study was based at a single institution from one country, which limits the generalizability of the findings. At our hospital, psychiatrists and obstetricians hold regular meetings to discuss the safe delivery and breastfeeding of pregnant women with psychiatric disorders, but this may not be the case at all hospitals. As another example, the prevalence of maternal depression is different among low‐, middle‐, and high‐income countries [43]. In order to clarify these points in the future, prospective studies using large samples under various circumstances, such as other areas in Japan and other countries, will be necessary.

In conclusion, taking psychotropic medications during the perinatal period may be safe and beneficial for pregnant women with psychiatric disorders and their children in terms of maintaining a better maternal psychiatric condition and newborn condition at birth and possibly during breastfeeding. Close cooperation between psychiatrists and obstetricians makes it possible for pregnant women with psychiatric disorders to give birth and breastfeed safely.

## Ethics Statement

This retrospective study was conducted according to the ethics committee of Ehime University Graduate School of Medicine (No. 2411002).

## Consent

The authors have nothing to report.

## Conflicts of Interest

The authors report no financial or other relationships relevant to the subject of this article. Jun‐ichi Iga is an Editorial Board member of Neuropsychopharmacology Reports and a co‐author of this article. To minimize bias, they were excluded from all editorial decision‐making related to the acceptance of this article for publication.

## Supporting information


Data S1.

## Data Availability

The data that supports the findings of this study are available in the Supporting Information of this article.
